# Drought-Induced Zinc Finger Transcription Factor OsDi19-3 Positively Regulates Drought Stress Acclimatization in Rice (*Oryza sativa* L.)

**DOI:** 10.3390/plants14101560

**Published:** 2025-05-21

**Authors:** Yanjie Li, Tianjiao Mu, Tianying Ren, Pan Li

**Affiliations:** 1 School of Chemistry and Chemical Engineering, Shandong University, Jinan 250100, China; 2 The Key Laboratory of Plant Development and Environment Adaptation Biology, Ministry of Education, School of Life Science, Shandong University, Qingdao 266237, China; 3 State Key Laboratory for Macromolecule Drugs and Large-Scale Manufacturing, School of Pharmaceutical Sciences and Food Engineering, Liaocheng University, Liaocheng 252059, China

**Keywords:** rice, OsDi19-3, zinc finger, drought, OsNEK6, OsCAMK1

## Abstract

The plant Di19 (drought-induced 19) protein belongs to zinc finger transcription factors, which play crucial roles in drought stress acclimatization. OsDi19-3, a drought-induced transcription factor in rice, has not been fully characterized for its biological role in stress acclimatization. In this study, transgenic rice overexpressing *OsDi19-3* was generated. Water deprivation experiments showed that transgenic plants exhibited higher drought tolerance than wild-type (WT) plants, indicating that OsDi19-3 positively regulates drought stress acclimatization. Consistent with this, stomata in overexpression lines closed more significantly than those in WT under drought stress. To explore the molecular mechanism, yeast two-hybrid and bimolecular fluorescence complementation (BiFC) experiments identified two interacting proteins of OsDi19-3: OsCAMK1 and OsNEK6. Notably, these two proteins also interacted with each other. A transcriptome analysis of *OsDi19-3* transgenic plants revealed 224 upregulated and 167 downregulated genes (log_2_(OE/WT) > 1, *p*-value < 0.05), including multiple stress-responsive genes. Furthermore, a ChIP-PCR analysis confirmed that OsDi19-3 directly binds to three target genes. This study provides insights into the role of OsDi19-3 in drought acclimatization and its regulatory network in rice.

## 1. Introduction

With global climate change and the increasing frequency of extreme weather events, drought has emerged as one of the most significant abiotic stressors, causing crop yield losses. Rice, a staple crop for over half of the world’s population, has particularly high water requirements throughout its growth stages. The reproductive stage is especially sensitive to water deficits, which severely affect both yield and grain quality.

The Di19 (drought-induced 19) family comprises small, conserved C2H2-type zinc finger transcription factors, widely distributed in plants. To date, their roles in stress responses have been characterized in numerous species, including Arabidopsis (*Arabidopsis thaliana*) [1,2], rice [3], cotton (*Gossypium hirsutum* L.) [4,5], maize (*Zea mays* L.) [6], wheat (*Triticum aestivum* L.) [7], poplar (*Populus trichocarpa* L.) [8], beans (*Phaseolus vulgaris* L.) [9], and *foxtail millet* (*Setaria italica* L.) [10]. Most *Di19* members are drought-inducible, which is consistent with their nomenclature [11]. Many Di19 proteins enhance drought acclimatization, while others act as negative regulators. For example, overexpression of *OsDi19-4* in rice improves drought tolerance [12], and ZmDi19-1 from maize confers drought resistance in transgenic Arabidopsis [6]. In poplar, homologous drought-induced 19 proteins, PtDi19-2 and PtDi19-7, could enhance drought tolerance in transgenic plants [8]. Conversely, *AtDi19-3* overexpression in Arabidopsis increases drought sensitivity [2]. The wheat TaDi19A exacerbates salt and osmotic stress susceptibility [7], and GhDi19-3 and GhDi19-4 in cotton also play negative roles in response to salt stress [5].

Di19 proteins also engage in diverse interaction networks, like some other transcription factors. In Arabidopsis, AtDi19-7 interacts with LKP2, a component of light-signaling pathways [12], while AtDi19-1 modulates auxin signaling via association with IAA14 [13]. In crops, OsDi19-5 binds OsClo5, suppressing salt-responsive genes [14]. SiDi19-3 interacts with SiPLATZ12 to enhance salt tolerance in foxtail millet [10]. GmDi19-5 from soybean (*Glycine max* L.) interacts with GmLEA3.1 and enhances the sensitivity of transgenic plants to abiotic stress [15]. Recently, Huang et al. (2024) also revealed the interaction of OsCactin with OsDi19, which positively regulates the drought stress response in rice [16].

Notably, emerging evidence indicates that multiple Di19 family members undergo phosphorylation by CPK/CDPK kinases [1]. For instance, AtDi19 is phosphorylated by AtCPK11, linking calcium signaling to drought responses [1]. Di19-2 could be phosphorylated by CPK16 in Arabidopsis [17]. Phosphorylation of AtDi19-3 by calmodulin-interacting kinase CIPK11 suppresses drought stress responses [18]. OsDi19-4 phosphorylation by calcium-activated OsCDPK14 enhances its transcriptional activity [3,19]. Cotton GhDi19-1/2 require CDPK-mediated phosphorylation for salt and drought stress adaptation [20].

The evolutionary conservation and functional diversity of Di19 proteins across plant species underscore their essential roles in stress acclimatization. To expand the functional characterization of plant Di19 members, we focused on OsDi19-3 in this study. Notably, we identified two novel interaction partners: a calcium/calmodulin-dependent protein kinase (designated CAMK1) and the rice NEK6 protein. Both CAMK1 and NEK6 participate in drought responses and demonstrate reciprocal interaction, revealing a previously uncharacterized regulatory mechanism within the Di19 family. Through integrated transcriptomic profiling and ChIP-PCR validation, we further identified three stress-responsive downstream targets of OsDi19-3, confirming its central regulatory role in stress signaling pathways. These discoveries significantly enhance our understanding of Di19 protein networks and establish new paradigms for drought stress regulation in plants.

## 2. Results

### 2.1. Characterization of OsDi19-3

**Figure 1:**
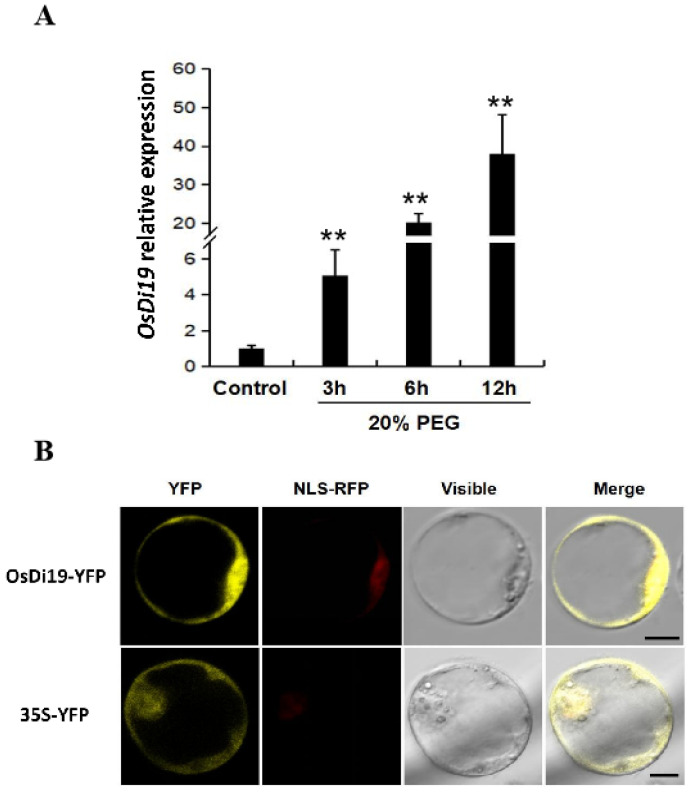
Characteristics of OsDi19-3. (**A**) Drought stress induction of *OsDi19-3* assessed by qPCR. For drought treatment, two-week-old WT rice plants were exposed to 20% PEG for 3 h, 6 h, and 12 h, and *OsDi19-3* expression was assessed by qPCR. Error bars represent standard deviation (SD) from three biological replicates. Asterisks indicate significant difference relative to control condition (Student’s *t* test, ** *p* < 0.01). (**B**) Subcellular localization of OsDi19-3 protein with NLS-RFP as nuclear localization signal; bar = 5 μm.

The rice gene *OsDi19-3* (*Os01g0672400*) encodes a Cys2/His2-type zinc finger protein comprising 246 amino acids. To investigate its drought responsiveness, two-week-old wild-type rice seedlings were treated with 20% (*w*/*v*) PEG-6000 to simulate drought response, and *OsDi19-3* expression was quantified by qRT-PCR at 0, 3, 6, and 12 h post-treatment. The results revealed a time-dependent progressive upregulation of *OsDi19-3* under drought stress (Figure 1A). A subcellular localization analysis was performed by amplifying the full-length coding sequence (CDS) of *OsDi19-3* and constructing a fusion expression vector with the fluorescent reporter gene driven by the 35S promoter, which was then introduced into rice protoplasts. Laser confocal microscopy demonstrated that OsDi19-3 exhibits dual localization in both the nucleus and cytoplasm (Figure 1B).

### 2.2. OsDi19-3 Functions as a Positive Regulator of Drought Stress Acclimatization

**Figure 2:**
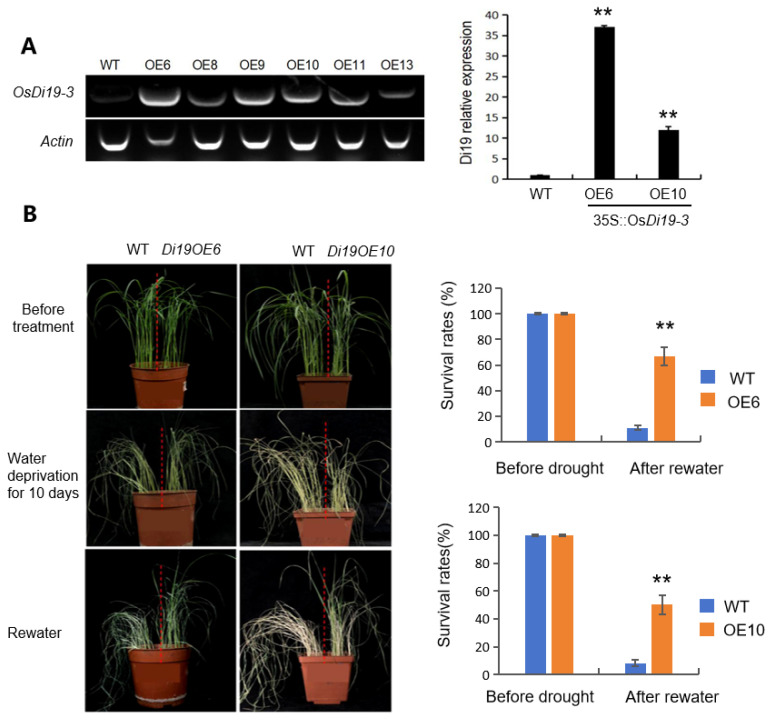
OsDi19-3 enhanced drought stress tolerance in the overexpression (OE) rice lines. (**A**) *OsDi19-3* expression levels evaluated by RT-PCR and quantitative PCR. (**B**) Drought-tolerant phenotypes and survival rates of *OsDi19-3*-overexpression lines OE6 and OE10 in comparison with wild type (WT). Asterisks indicate significant difference relative to WT (Student’s *t* test, ** *p* < 0.01).

To investigate the biological role of OsDi19-3 in drought resistance, we generated transgenic rice lines overexpressing *OsDi19-3*, yielding two independent overexpression lines (OE6 and OE10) with significantly elevated transcript levels (Figure 2A).

Under progressive drought stress of three-week-old plants subjected to water withholding for 21 days, wild-type (WT) plants exhibited severe wilting and chlorosis, whereas the OE6 and OE10 lines maintained turgor and green foliage (Figure 2B). Following rehydration, the survival rates of OE6 (66.7%) and OE10 (50.1%) significantly surpassed those of WT plants (11.1% and 8.3%, respectively) (Figure 2B), confirming enhanced drought recovery capacity in *OsDi19-3*-overexpressing plants.

**Figure 3:**
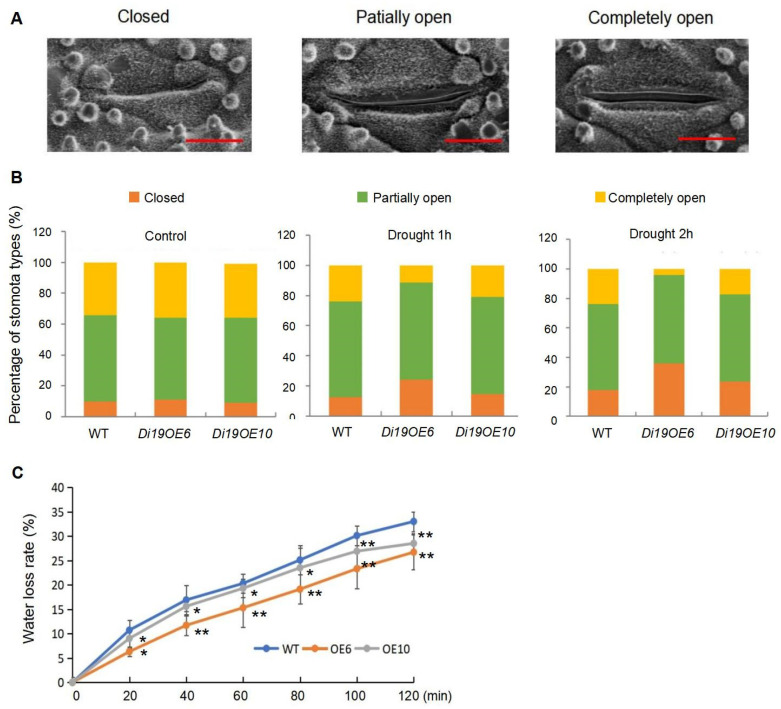
Overexpression of *OsDi19-3* promotes stomatal closure in rice. (**A**) Scanning electron microscopy images of three levels of stomatal apertures. Bar: 5 μm. (**B**) The percentage of three levels of stomatal aperture in the leaves of *OsDi19-3*-overexpression lines and WT rice plants under normal and drought conditions. (**C**) Water loss rates of the detached leaves from *OsDi19-3* transgenic plants and wild type. Asterisks indicate significant difference relative to WT (Student’s *t* test, * *p* < 0.05, ** *p* < 0.01).

Drought-induced stomatal closure, a key mechanism to mitigate water loss via reduced transpiration, was further analyzed. Quantification of stomatal aperture states (closed, semi-open, fully open) under air-drying conditions revealed distinct patterns (Figure 3A). Under non-treatment condition, no significant differences in stomatal status were observed between WT and transgenic lines. When subjected to 1 h of air-drying, the closed stomata proportions increased to 24.0% (OE6) and 14.5% (OE10) versus 12.4% (WT), while fully open stomata decreased to 11.1% (OE6) and 20.9% (OE10) compared to 23.9% (WT) (Figure 3B). When subjected to 2 h of air-drying, closed stomata further increased to 36.0% (OE6) and 23.4% (OE10) versus 17.9% (WT), with fully open stomata falling to 3.9% (OE6) and 17.2% (OE10) relative to 23.6% (WT) (Figure 3B).

Consistent with these observations, detached leaves from OsDi19-3-overexpressing plants displayed significantly reduced water loss rates compared to WT under dehydration stress (Figure 3C). Collectively, these data demonstrate that OsDi19-3 enhances drought tolerance by promoting stomatal closure and limiting transpirational water loss.

### 2.3. *OsDi19-3* Interacts with *OsCAMK1* and *OsNEK6* Proteins

**Figure 4:**
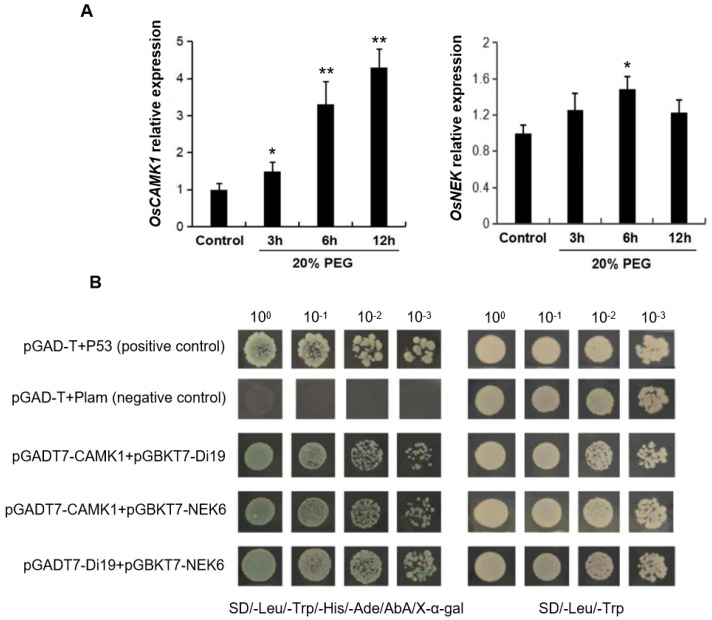
Identification of OsDi19-3-interacting proteins. (**A**) Drought stress response of *OsCAMK1* and *OsNEK6*. Asterisks indicate significant difference relative to WT (Student’s *t* test, * *p* < 0.05, ** *p* < 0.01). (**B**) Interaction of OsDi19-3, OsNEK6, and OsCAMK1 identified by yeast two-hybrid. The full-length cDNAs of OsDi19-3, CAMK1, and NEK6 were cloned into pGBKT7 (bait) or pGADT7 (prey). The derived prey and bait constructs were co-transformed into the yeast strain Y2H Gold. The transformed yeast cells were plated onto the SD/-Leu/-Trp/-His/-Ade/AbA/X-gal medium (left) and SD/-Leu-Trp medium (right).

To elucidate the upstream regulatory mechanisms of OsDi19, we employed its N-terminal sequence as bait in a yeast two-hybrid screen against a rice cDNA library. This screening yielded two positive clones, which were subsequently sequenced and identified as (1) a novel calcium/calmodulin-dependent protein kinase, designated OsCAMK1 (Os03g0122000); and (2) a partial coding sequence corresponding to the rice NIMA-related kinase 6 (OsNEK6, Os02g0590800). NIMA-related kinase (NEK), belonging to a family of serine/threonine kinases, is mainly involved in cell-cycle processes in plants [21]. To assess their drought stress responsiveness, we conducted a qRT-PCR analysis. The results demonstrated significant transcriptional upregulation of *OsCAMK1* under drought stress conditions, whereas *OsNEK6* exhibited no statistically significant induction at the transcript level (Figure 4A). Notably, despite this lack of transcriptional response, OsNEK6 has been previously implicated in drought stress regulation [22]. To validate protein–protein interactions, we performed yeast two-hybrid assays. The full-length coding sequence (CDS) of *OsDi19-3* was cloned into both the pGBKT7 (bait) and pGADT7 (prey) vectors, while OsCAMK1 and OsNEK6 were independently cloned into pGBKT7. Co-transformation experiments revealed robust growth of yeast cells harboring pGADT7-OsDi19-3 paired with pGBKT7-OsCAMK1 or pGBKT7-OsNEK6 on selective media (Figure 4B). Intriguingly, reciprocal co-transformation of pGADT7-OsCAMK1 with pGBKT7-OsNEK6 also supported yeast growth, suggesting a direct interaction between OsCAMK1 and OsNEK6 independent of OsDi19-3. These findings collectively indicate potential interactions among OsDi19-3, OsCAMK1, and OsNEK6.

**Figure 5:**
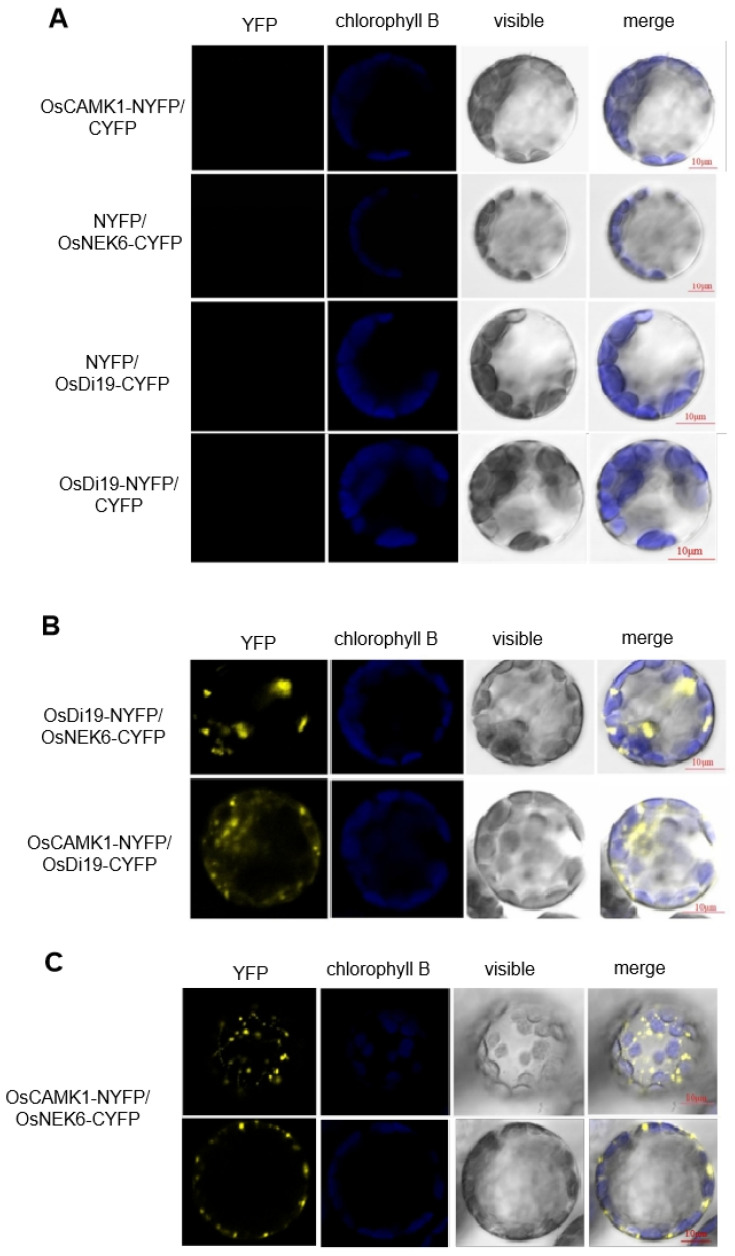
Interaction of OsDi19-3, OsNEK6, and OsCAMK1 identified by BiFC. (**A**) Signal visualization when OsCAMK1-NYFP, OsNEK-CYFP, OsDi19-CYFP, and OsDi19-NYFP were co-transformed with control vector. (**B**) Signal visualization when OsDi19-NYFP/OsNEK6-CYFP and OsCAMK1-NYFP/OsDi19-CYFP were co-transformed. (**C**) Signal visualization when OsCAMK1-NYFP/OsNEK6-CYFP were co-transformed. Bar = 10 μm.

To further corroborate these interactions, bimolecular fluorescence complementation (BiFC) assays were conducted in rice protoplasts. The constructs OsCAMK1-nYFP and OsDi19-cYFP, OsDi19-nYFP and OsNEK6-cYFP, and OsCAMK1-nYFP and OsNEK6-cYFP were co-transfected and analyzed via confocal microscopy. Distinct fluorescence signals were observed in protoplasts co-expressing each protein pair (Figure 5), whereas control experiments with empty vectors showed no detectable fluorescence. These results confirmed pairwise interactions among OsDi19-3, OsCAMK1, and OsNEK6, supporting the formation of a ternary protein complex.

### 2.4. Transcriptomic Profiling of OsDi19-3 Overexpression Lines Reveals Stress-Responsive Regulatory Networks

**Figure 6:**
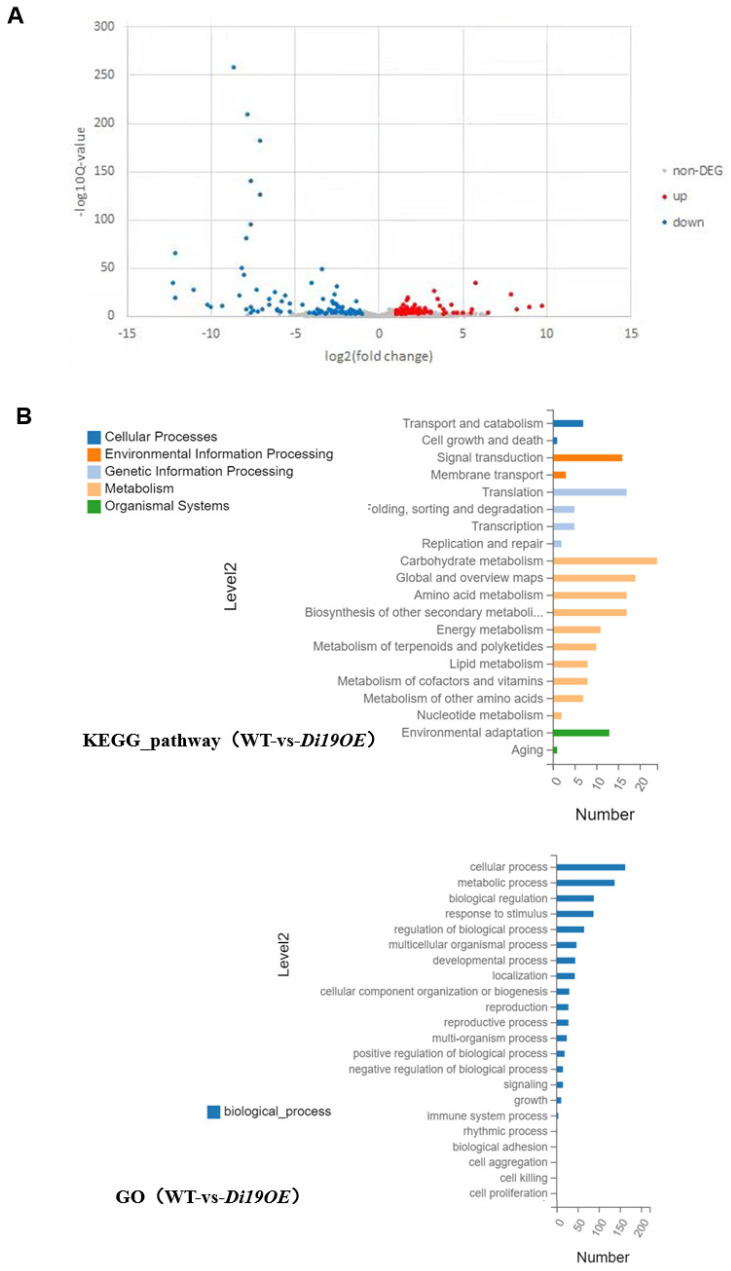
Transcriptome profiling of *OsDi19-3* overexpression lines. (**A**) Up- and downregulated genes upon *OsDi19-3* overexpression. Differentially expressed genes classified via KEGG and GO analysis (**B**).

The functional characterization of OsDi19-3 as a drought-responsive transcription factor prompted investigation into its downstream regulatory targets. To delineate its molecular mechanisms, we performed RNA sequencing on *OsDi19-3* overexpression (OE) lines, identifying 224 significantly upregulated (log_2_(OE/WT) > 1, *p* < 0.05) and 167 downregulated transcripts compared to wild-type (WT) controls (Figure 6A).

Gene Ontology (GO) enrichment and Kyoto Encyclopedia of Genes and Genomes (KEGG) pathway analyses revealed pronounced enrichment of biological processes related to response to abiotic stimulus and environmental acclimatization (Figure 6B), corroborating the central role of OsDi19 in stress tolerance.

Cross-referencing differentially expressed genes (DEGs) with the Genevestigator transcriptomic database demonstrated significant overlap with abiotic stress-responsive modules, particularly those regulated by drought (46.3%), salinity (32.1%), and heat stress (21.6%) (Table 1). Notably, validated stress-associated genes were identified among the DEGs. For instance, Shim et al. (2023) recently reported OsDIAT (Os05g0244700), an aminotransferase IV family member, could enhance drought acclimatization [23]. OsHKT9 (Os06g0701600), a sodium transporter, was found to play a pivotal role in salt tolerance [24]. In addition, OsUGT3 (Os02g0755900), a cytokinin-O-glucosyltransferase, was reported to increase drought and salt tolerance through modulating ABA synthesis and scavenging ROS in rice [25].

**Figure 7:**
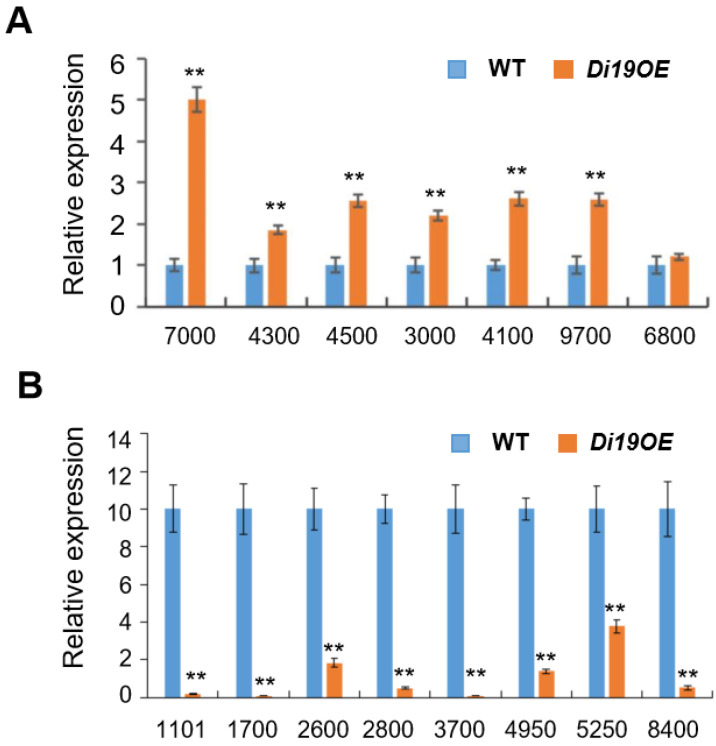
QPCR verification of the upregulated (**A**) and downregulated (**B**) genes in *OsDi19* OE6 plants identified in transcriptome profiling. RNA was prepared from three-week-old OE6 transgenic plants. Error bars represent standard deviation (SD) from three biological replicates. Asterisks indicate significant difference relative to WT (Student’s *t* test, ** *p* < 0.01).

The transcriptional activation of *OsDi19-3* itself (log_2_(OE/WT) = 6.8) confirmed successful transgene overexpression in the profiling system. To validate sequencing reliability, we conducted qRT-PCR on independent biological replicates using three-week-old WT and *OsDi19*-OE6 seedlings. Six of the seven selected upregulated genes—*Os11g0707000*, *Os01g0314300*, *Os01g0584500 Os07g0163000*, *Os11g0694100*, and *Os04g0649700*—exhibited significant upregulation, while *Os06g0116800* showed marginal upregulation (Figure 7). Conversely, all eight downregulated candidates—*Os07g0561101*, *Os04g0381700*, *Os11g0262600*, *Os01g0952800*, *Os03g0293700*, *Os06g0294950*, *Os10g0195250*, and *Os07g0258400*—displayed significant suppression in OE lines (Figure 7). These orthogonal validations confirm the robustness of our transcriptomic dataset.

### 2.5. Identification of OsDi19-3 Downstream Target Genes

The transcriptome analysis demonstrated significant differential expression of stress-responsive genes in OsDi19-3-overexpression lines. To elucidate the direct regulatory targets of this transcription factor, we performed cis-element analysis on their upstream promoter regions. Based on previous reports of the OsDi19-3 binding motif TACAA(G)T [26], we systematically screened the promoter regions (−800 bp) of the top 156 differentially expressed genes (log_2_(OE/WT) > 1.5). This screening identified 12 candidate genes containing the characteristic motif (Table 2).

**Figure 8:**
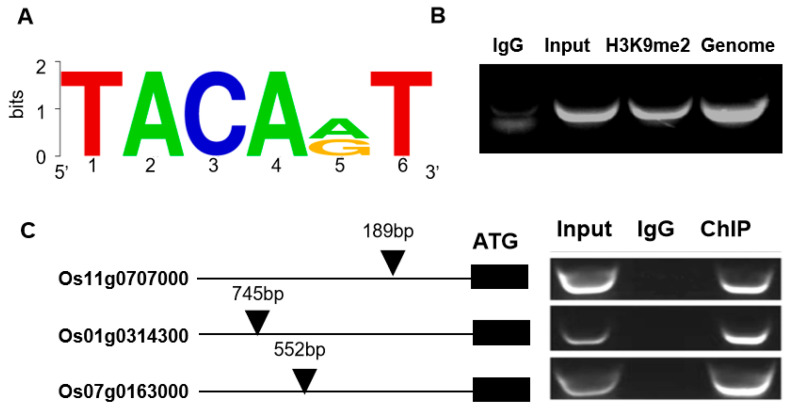
Identification of Di19-3 target genes by ChIP-PCR. (**A**) Di19-3 binding motif. (**B**) Control gene amplified with IgG-precipitated sample (negative control), input, H3K9me2-precipitated sample (positive control), and rice genome. (**C**) Promoter fragments of the target genes amplified from FLAG-precipitated sample with three-week-old OE6 transgenic plants.

To validate direct binding, chromatin immunoprecipitation (ChIP) assays were conducted using 35S::*OsDi19*-FLAG transgenic plants, with specific primers designed to amplify sequences flanking the putative binding motifs. Successful amplification was achieved for three genes: Os11g0707000 (Rubisco activase, AAA+ family), Os01g0314300 (D-lactate dehydrogenase), and Os07g0163000 (translation machinery-associated protein) (Figure 8C). Consistent with the ChIP results, these genes exhibited remarkable upregulation in transcriptome data, with log_2_(OE/WT) values of 27.93, 21.28, and 7.64, respectively, confirming the possibility of their direct transcriptional regulation by OsDi19. Notably, functional annotation of the three genes revealed significant biological relevance to stress acclimatization. For instance, Os11g0707000 encodes a Rubisco activase documented to mediate stress acclimatization [27,28]. Although Os01g0314300 and Os07g0163000 have not been functionally characterized, their responses to stresses are shown in the public transcriptome datasets in RAP-DB (https://rapdb.dna.affrc.go.jp/, accessed on 30 January 2025), both of which are induced by salt and drought.

## 3. Discussion

The plant Di19 protein family has attracted increasing research interest due to its diverse roles in plant biology. Emerging evidence suggests that Di19 family members participate not only in abiotic stress acclimatization but also in other physiological processes. Systematic characterization of more Di19 homologs could significantly advance our understanding of plant stress signaling networks.

The rice genome contains seven Di19 genes, yet only OsDi19-4 and OsDi19-5 have been functionally characterized [3,14]. Despite sharing transcription factor classification within rice, these two proteins demonstrate distinct stress response patterns and biological roles, revealing functional specialization within the Di19 family through evolutionary divergence. This study focuses on exploring the role of OsDi19-3, a previously uncharacterized rice Di19 homolog. While earlier work documented drought- and salt-induced expression patterns of OsDi19-3, its functional relevance remained unexplored [3]. We specifically investigated its role in drought stress response through transgenic overexpression. Our observations revealed that OsDi19-3-overexpressing rice lines exhibited enhanced drought tolerance, demonstrating its positive regulatory function in drought acclimatization. This finding contrasts with several Di19 homologs in stress responses, including Arabidopsis AtDi19-3 [2], wheat TaDi19A [7], and cotton homologs GhDi19-3/GhDi19-4 [5], which were upregulated by abiotic stresses, but play negative regulatory roles in stress acclimatization. Such functional divergence highlights the complexity of Di19-mediated stress regulation across plant species. Transcriptome profiling of OsDi19-3-overexpression lines revealed numerous stress-regulated differentially expressed genes (DEGs) involved in diverse physiological processes. Phylogenetic analysis showed that OsDi19-3 is most closely related to OsDi19-4, with both genes enhancing drought tolerance. However, OsDi19-4 primarily regulates drought stress through the ABA signaling pathway, as evidenced by its two ABA-related targets and the enrichment of ABA signaling pathway genes in its transcriptomic analysis [19]. In contrast, we detected few ABA-related pathway genes in the OsDi19-3 transcriptome, suggesting that OsDi19-3 employs a different stress regulation mechanism from OsDi19-4.

Numerous Di19 family members have been reported to undergo phosphorylation by CPK/CDPK kinases, a process that potentially facilitates nuclear translocation and enhances their transcriptional activity under stress conditions [1]. A subcellular localization analysis demonstrated that OsDi19-3 exhibits dual localization in both the cytoplasm and nucleus (Figure 1B). This bipartite localization pattern is evolutionarily conserved among various Di19 family members, including OsDi19-7 in rice, AtDi19-2 in Arabidopsis, and ZmDi19-1 in maize [3,6,11]. Such localization features suggest potential interactions with cytoplasmic protein kinases that could mediate post-translational modifications and subsequent nuclear translocation. Supporting this mechanism, Fan et al. (2023) reported that AtCPK12 undergoes calcium-dependent phosphorylation during hypoxia, triggering its cytoplasmic-to-nuclear translocation [29].

In this study, we also tried to identify proteins interacting with OsDi19-3, and found two kinase interactors of OsDi19-3: OsCAMK1, a previously uncharacterized calcium/calmodulin-dependent kinase; and OsNEK6, a Ser/Thr-type NIMA-related kinase with established drought stress associations [28,29,30]. Notably, both of them exhibit functional links to drought acclimatization. Intriguingly, OsDi19-3 interacts independently with both kinases (Figure 4B and Figure 5), while mutual binding of OsCAMK1 and OsNEK6 was also observed, a novel interaction triad among Di19 proteins. This suggests a potential phosphorylation-mediated regulatory mechanism through ternary complex formation. Prior studies identified diverse Di19 interactors [10,13,16,21], whereas our work provides the first evidence of a three-component interaction network involving Di19 and functional crosstalk between calcium-dependent and cell-cycle-related kinases in Di19-mediated regulation.

NEK proteins are evolutionarily conserved regulators of mitotic progression and chromosomal dynamics [30,31,32]. In plants, NEK genes tend to exhibit microtubule-associated localization patterns, as demonstrated by the co-localization of GmNEK1 (soybean) and OsNEK6 (rice) with tubulin markers, which showed spotted localization patterns [22,33]. Consistently, in our BiFC results (Figure 5B,C), the co-localization of Di19, OsCAMK1, and OsNEK6 also exhibited similar patterns, suggesting that they might be associated with the microtubule. NEKs were also identified to be involved in the plant stress pathways. For instance, Arabidopsis NEK enhances salinity and osmotic stress tolerance [21]. Soybean *NEK* family members (*NEK1*, *5*, *6*, *7*, *9*, *13*, *14*, *15*) show significant induction under salt, drought, and cold stress [33]. Ning et al. (2011) found that rice SINA E3 ligase-mediated degradation of OsNEK6 reduces drought tolerance, implying its positive regulatory role [22]. Our results extend this finding by revealing a functional link between OsNEK6 and drought acclimatization. We propose that SINA E3 ligase-mediated degradation of OsNEK6 may disrupt its interaction with Di19-3, potentially attenuating drought stress responses. This hypothesis aligns with and mechanistically refines previous observations of the drought tolerance of OsNEK6.

As stress-responsive transcription factors, Di19 proteins confer environmental adaptability through direct regulation of downstream targets. For example, AtDi19 in Arabidopsis binds promoters of the drought-responsive genes PR1, PR2, and PR5 to coordinate drought acclimatization [26]. OsDi19-4 activates the ABA-responsive genes OsASPG1 and OsNAC18 via promoter binding [19]. OsDi19-5 suppresses the salt-stress inducible genes OsUSP and OsMST [14]. To elucidate the transcriptional network governed by OsDi19-3, we integrated transcriptome profiling and cis-element analysis. Transcriptome sequencing revealed that a large number of stress-related DEGs were enriched, participating in various abiotic stress pathways, which highlights the roles of OsDi19-3 involved in not only drought but also other abiotic stress regulation.

Promoter motif screening identified that the conserved TACA(A/G)T binding signature of Di19 exists in many DEGs. Finally, ChIP validation confirmed direct binding to three drought-responsive targets in this work. Of these, Os11g0707000 encodes a Rubisco activase (RCA), which is critical for Rubisco activation and stress acclimatization [28,34]. Rubisco activase (RCA) catalyzes the release of inhibitory sugar phosphates from Rubisco, and might be a potential strategy for improving a photosynthesis-driven increase in crop yield [35,36]. This study primarily reveals the regulation of a Rubisco activase by OsDi19-3, and will be highly significant, as this pathway not only enhances plant drought tolerance but also holds substantial research potential for improving crop yield under drought conditions, which is worthy further studying.

The other two are novel target genes. Notably, all three targets exhibited significant upregulation in transgenic lines, consistent with the role of OsDi19-3 as a transcriptional amplifier. These findings establish its critical role in drought signaling. Of course, we believe OsDi19-3 surely has other target genes that remain to be identified. The full regulatory scope of OsDi19-3 likely extends beyond these validated targets, as suggested by transcriptome-wide modulation of stress-related genes.

## 4. Conclusions

Our study advances understanding of OsDi19-3 through two key aspects. First, we observed the interaction network of the CAMK1-NEK6-Di19-3 ternary complex, which redefines Di19-mediated signaling and reveals unprecedented crosstalk between calcium-dependent and cell-cycle-associated kinases. Second, the discovery of drought-responsive targets establishes OsDi19-3 as a transcriptional factor coordinating drought acclimatization. 

These investigations will bridge the gap between mechanistic understanding and agronomic application, positioning OsDi19-3 as both a scientific paradigm and a biotechnological tool for climate-smart agriculture. Further investigation of this signaling pathway may not only reveal the mechanism of Di19-3 in coping with drought stress, but also contribute to crop breeding in resisting stress conditions, which will be the focus of our further research.

## 5. Materials and Methods

### 5.1. Plant Materials and Growth Conditions

All experiments utilized Oryza sativa subsp. japonica cv. Zhonghua 11 (ZH11), with transgenic lines generated in the ZH11 genetic background. Rice seed sterilization was performed by treating with 75% alcohol for 1 min, and then shaking in a solution containing 10% sodium hypochlorite, followed by 2% Tween-20 for 25 min, and finally washing five times with sterile water for 1 min each time. Seeds were cultured on MS medium for about 10 days and then transplanted to planting pots with soil for further cultivation at 23 °C.

### 5.2. Determination of the Subcellular Localization

The full-length coding sequence OsDi19-3 ORF was PCR-amplified and directionally cloned into the PAN581-35S-YFP binary vector to generate the 35S::OsDi19-3-YFP fusion. The fusion constructs were then transformed into rice protoplasts, and transiently expressed for 16–18 h in dark. Meanwhile, NLS-RFP was used as the nuclear localization marker. The fluorescent signals were taken and observed by a Zeiss LSM 880 Airyscan (Jena, Germany).

### 5.3. Gene Cloning, Vector Construction, and Plant Transformation

The gene sequence information of the rice was obtained from The Rice Annotation Project Database (https://rapdb.dna.affrc.go.jp/download/irgsp1.html, accessed on 1 May 2022). For generating OsDi19-3 transgenic rice plants, OsDi19-3 (Os01g0672400) CDS was PCR-amplified and sequenced to be correct. Then, the fragment was inserted into the pUN1301 binary vector under control of the CaMV 35S promoter. The construct was introduced into ZH11 rice by agrobacterium-mediated transformation and the T_0_-positive plants were selected with hygromycin. The transformation was performed by Biorun biological Co., Ltd. (Wuhan, China).

### 5.4. Electron Microscopy Scanning for Stomatal Aperture

Treated or untreated 3-week-old rice leaves were detached and kept in air for one or two hours to allow water loss and stimulate stomatal closure. Then they were cut into 0.5 cm × 0.5 cm size squares with a sharp blade and immediately immersed in 2.5% glutaraldehyde, fixed at 4 °C for 3 h, and then the supernatant was discarded. Subsequently, the sample was washed three times with 1×PBS. The sample was then dehydrated with ethanol aqueous solutions in a gradient of concentrations of 30%, 50%, 70%, 80%, and 90% for about 15 min each time. The supernatant was discarded and the sample was dehydrated twice with anhydrous ethanol. After the sample was fully dried, it was sputtered with gold and observed under a scanning electron microscope (FEI Quanta250 FEG, OR, USA). For each line, at least 100 stomata were observed.

### 5.5. Yeast Two-Hybrid and BiFC Experiment

For the yeast two-hybrid assay, the ORFs of OsDi19-3 and NEK6 genes were cloned into the pGBKT7 vector. The ORFs of OsDi19-3 and CAMK1 were each inserted into the pGADT7 vector. Then, these constructs, in pairs, were co-transformed into yeast strain Y2H Gold, followed by plating onto SD/-Trp/-Leu media. After growing for 3 days, the clones were tested for protein interactions by transferring them onto SD/-Ade/-His/-Trp/-Leu/X-α-Gal plates for 3–5 days.

For the BiFC experiment, various constructs in the vectors pSAT6-NYFP or pSAT6-CYFP were transiently expressed in rice protoplasts via PEG transformation for 2 days, and the fluorescence was checked by a Zeiss LSM 880 Airyscan (Jena, Germany).

### 5.6. Transcriptome Profiling

Three-week-old Di19OE6 and WT rice seedlings with good growth status and consistent growth were obtained as materials for transcriptome analysis. They were rapidly frozen in liquid nitrogen and sent to BGI (Beijing, China) for transcriptome sequencing. Three biological replicates were performed in this analysis.

### 5.7. Quantitative Real-Time PCR

For quantitative PCR, RNA was extracted from rice samples with the Trizol reagent (Vazyme, Nanjing, China), and 5 µg of RNA was reverse-transcribed with the PrimeScript RT reagent kit with gDNA Eraser (Vazyme) according to the supplier’s manual. Quantitative PCR was performed with a real-time thermal cycling system (Bio-Rad, CA, USA). SYBR-Green was used to detect gene abundances. Three biological replicates of each reaction were performed. The data were analyzed using the Bio-Rad CFX Manager software 1.1. Expression levels were normalized with the reference gene Actin 2. The PCR reactions were performed in a 25 µL total reaction volume, with 25 cycles for amplifying OsActin2 and 32 cycles for amplifying the detected genes. The primer information for the qPCR assay is included in Table S1.

### 5.8. ChIP-PCR Analysis

Three-week-old 35S::*OsDi19*-FLAG transgenic plants were used for the ChIP assay using the EpiQuik™ Plant ChIP Kit (EpiGentek, NY, USA). PCR was used to detect the amount of precipitated DNA and input DNA. Promoter primers were designed to amplify a fragment length ranging between 100 and 150 bp, and locating within 800 bp upstream sequence from the ATG. The relevant primers are given in Table S1.

## Figures and Tables

**Table 1:** Upregulated stress-responsive genes in *OsDi19-3*-overexpression plants.

Gene ID	Log_2_ (Di19OE/WT)	Gene Name/Functional Description	Stress Regulation ^a^
Os11g0707000	27.93	Rubisco activase	Heat, salt, drought
Os01g0314300	21.18	Translation machinery-associated protein	Not available
Os01g0800300	20.68	Unknown	Not available
Os06g0116800	8.41	OsDjA9	Biotic stress
Os05g0244700	7.97	Aminotransferase, OsDIAT	Drought
Os07g0163000	7.64	D-lactate dehydrogenase	Not available
Os07g0633100	7.55	X8 domain containing protein	Drought
Os01g0672400	6.81	OsDi19-3	Drought, salt
Os01g0692400	4.96	Unknown	Drought
Os05g0317900	3.81	OsCrRLK1L2	Abiotic and biotic stress
Os08g0496700	3.64	Myb/SANT-like domain containing protein	Not available
Os11g0694100	3.39	OsWAK123	Biotic and abiotic stress
Os09g0396900	2.87	Vacuolar Iron Transporter 2	Drought
Os04g0677300	2.86	Harpin-induced protein 1 domain-containing protein	Drought
Os04g0584500	2.66	OsPUB77, OsEnS-73	Drought
Os01g0638000	2.51	Anthocyanin 3-O-beta-glucosyltransferase	Drought
Os01g0635200	2.40	MYB family transcription factor	Abiotic stress
Os01g0227800	2.39	cytochrome P450	Drought
Os01g0227100	2.22	NON-YELLOW COLORING 1	Drought
Os03g0218400	2.21	MONOSACCHARIDE TRANSPORTER 4	Drought
Os04g0447700	2.07	Oxidoreductase, aldo/keto reductase family protein	Abiotic stress
Os02g0755900	1.97	Cytokinin-O-glucosyltransferase, OsUGT3	Drought
Os01g0200700	1.92	Metallothionein-like protein	Tolerant to salinity and heavy metal stress
Os06g0701600	1.90	Na+ and K+ transport, OsHKT9	Salt tolerance
Os01g0822800	1.84	RING-H2 finger protein	Drought
Os11g0106700	1.80	Ferritin 1	Drought
Os03g0268600	1.75	Protein phosphatase 48	Drought
Os05g0373900	1.71	Unknown	Drought
Os01g0160800	1.63	RIBOSOME-INACTIVATING PROTEIN 1.1	Drought
Os12g0106000	1.60	FERRITIN 2	Drought
Os04g0632100	1.59	S-Domain kinase-7	Drought
Os04g0684800	1.52	OsUEV1D	Abiotic stress
Os03g0729000	1.50	Peptidase	Drought

^a^: Stress regulation information is from GO analysis, Genevestigatior, or publications.

**Table 2:** Locus of Di19-binding element in upstream regions of some differentially expressed genes.

Gene ID	Log_2_ (Di19OE/WT)	Gene Name/Functional Description	Locus of TACAA(G)T from the ATG (bp)
Os11g0707000	27.93	Rubisco activase	−189, −318, −755
Os01g0314300	21.28	Uncharacterized	−745
Os01g0606400	9.70	Uncharacterized	−219, −339, −430
Os07g0163000	7.64	D-lactate dehydrogenase	−552
Os09g0560700	7.37	MOSC domain-containing protein	−253, −540
Os03g0218400	2.21	MONOSACCHARIDE TRANSPORTER 4	−521, −584
Os06g0701600	1.90	OsHKT9	−166, −536, −592
Os10g0502400	1.88	Glutamyl-tRNA reductase 2	−615
Os03g0268600	1.75	Protein phosphatase	−229
Os04g0632100	1.60	S-Domain kinase-7	−248
Os10g0209700	1.54	Heavy-metal-associated isoprenylated plant protein 28	−317
Os04g0684800	1.52	OsUEV1D	−290, −304, −612

## Data Availability

The data presented in this study are available in the paper and Supplementary Files.

## Supplementary Materials

The following supporting information can be downloaded at: https://www.mdpi.com/article/10.3390/plants14101560/s1, Table S1. Primers used in this study.
