# Supplementary material for: Drought-Induced Zinc Finger Transcription Factor OsDi19-3 Positively Regulates Drought Stress Acclimatization in Rice (Oryza sativa L.)
Source: Plants (Basel). 2025 May 21;14(10):1560. doi: 10.3390/plants14101560 (PMC12115221; doi:10.3390/plants14101560)
Supplement: Supplementary file 1 [file plants-14-01560-s001.zip › plants-3616663-supplementary.pdf]

Table S1. Primers used in this study.

Primer name	Forward (5'-3')	Reverse (5'-3')
**NEK6-BK**	GGCCATGGAGGCCGAATTCATGGAGCAGTACGAGGTGGTG	CGGATCCCCGGGAATTCCTACGTAAGTTTTGGTGACCCTTC
**NEK-AD**	ATGGAGGCCAGTGAATTCATGGAGCAGTACGAGGTGGTG	CCCACCCGGGTGGAATTCCTACGTAAGTTTTGGTGACCCTTC
**NEK-NYFP**	CGAGCTCAAGCTTCGAATTCTATGGAGCAGTACGAGGTGGTG	GGTACCGTCGACTGCAGAATTCTACGTAAGTTTTGGTGACCCTTC
**NEK-CYFP**	CGAGCTCAAGCTTCGAATTCTATGGAGCAGTACGAGGTGGTG	GGTACCGTCGACTGCAGAATTCTACGTAAGTTTTGGTGACCCTTC
**CAMK1-BK**	GGCCATGGAGGCCGAATTCATGACGATGGCTGCGGCGC	CGGATCCCCGGGAATTCTTAGTTCTGGACATATCTCTCCTGT
**CAMK1-AD**	ATGGAGGCCAGTGAATTCATGACGATGGCTGCGGCGC	CCCACCCGGGTGGAATTCTTAGTTCTGGACATATCTCTCCTGT
**CAMK1-MYC**	TTCTGCAGGGGCCCGGGGATGACGATGGCTGCGGCGC	TCCACTAGTATTTAAATGGTTCTGGACATATCTCTCCTGT
**CAMK1-CYFP**	CGAGCTCAAGCTTCGAATTCTATGACGATGGCTGCGGCGC	GGTACCGTCGACTGCAGAATTTTAGTTCTGGACATATCTCTCCTGT
**CAMK1-NYFP**	CGAGCTCAAGCTTCGAATTCTATGACGATGGCTGCGGCGC	GGTACCGTCGACTGCAGAATTTTAGTTCTGGACATATCTCTCCTGT
**CAMK1-YFP**	GGAGGAGCTGTACAGATCTATGACGATGGCTGCGGCGC	AGCCGGGCGGCCGCTTTAATTAGTTCTGGACATATCTCTCCTGT
**1300-OSDI19**	GAGAGAACACGGGGGACTATGGACTCGGAGCACTGGATC	CTTGTAGTCCATGTCGACGTCTTCAAATAAAGTGGAGAG
**DI19-YFP**	GGAGGAGCTGTACAGATCTATGGACTCGGAGCACTGG	AGCCGGGCGGCCGCTTTAATCAGTCTTCAAATAAAGTGGAG
**DI19-NYFP**	CGAGCTCAAGCTTCGAATTCTATGGACTCGGAGCACTGG	GGTACCGTCGACTGCAGAATTTCAGTCTTCAAATAAAGTGGAG
**PRO-3000**	GTTTGAAATTGTGAATTTGGAT	TTTGGGACGGAGGGAG
**PRO-4300**	TGCACTACCTCCCTCC	GCCTATCGCAGTCTCG
**PRO-9700**	GCAACAGTGACACCTTGTATCTTC	CTGTGCAGAATCTTTCTTTCTTTCC
**PRO-6400**	ACTGGGCAATAAGCTTCATATCATC	TTTAAAATGCTCTTGTTAGGGA
**PRO-4800**	GATGTGCAAGTCCAACCCTCAAAT	CCTTTTCGTTCGTCGGTGCC
**PRO-1600**	TTGGAAGAAGTGACTAGAGTCTAT	ACTTTACGAAACTACTAATGTGTAGTA
**PRO-2400**	CTCGCATTTCGCTCTTTGGGG	CCAGCATGTCACCGTTTGTCA
**PRO-2100**	CGCTCTCTTGCCAACTTTTCACG	GAATTTAGTACGGTGGGCCAACG
**PRO-8600**	AACGCACGCTGACGGCAAAGCTT	CCAAACATGCCCTTGGATTG
**PRO-7000**	GGGTGAATTCTGTGGTGAGGAA	GCCTTTTGCTGCCTCTCGTTGC
**PRO-0700**	CCTATGATAAGTACTCCATCTGTCTC	CAGTGCCAGTGTGGTGTGCAT
**PRO-8400**	GGACCAGGCTTTATGTGGAGA	ACCACTCCAAGATTAGCCGA
**qRT-DI19**	GCTTTCTTCGTTTGGCCTGG	CTCGTGAGCGATGAGTCGAT
**qRT-CAMK1**	AGGCATATGACCGTGCAGAG	GAGAGAGACTGGTGATTTC
**qRT-NEK6**	GCAGTGGCAGAAAGCGAATC	CTTGTGAAATGCAGGCGTCC
**qRT-0300**	GTGATCATAGCCTACGCGCT	TGCATCAGATCGGCAGAGAC
**qRT-2100**	GGAGGCTCAGAACCAAGAGG	GATGAGCTCCTCCCGCATC
**qRT-2600**	GCCGCTCATCAATCACGTTG	GCCTGAGAGCAGATCGACTT
**qRT-2800**	ATGTGGCGGGTTCAGTTCTT	CACAAAATGCTGGGCACTCC
**qRT-3000**	CACAAGGCAGTCAATGTGCC	AAACACCACCATGAGGTGCC
**qRT-3700**	CACAGGCAGGACATGGAGAG	TGTCCTTGTGATCTACGGCG

**qRT-4300**	GTTGGTTTCTTTCTTTAGCCAAAAT	TCCAAGGAACCACCTGGAGA
**qRT-4500**	TGCATCAGGGCAGAAGGAAG	ACGAACAGGAGACAGAACCT
**qRT-4950**	CGGTGTCACCATAGCTCCAG	GATGGGGTTGGTTGGGTTCA
**qRT-5250**	AGGCAATCAATCCTTCCCCG	TAGGGGCCAACAGGCCAAA
**qRT-7000**	GGGTGAATTCTGTGGTGAGGAA	GCCACACGCTGAAGATTGGG
**qRT-7300**	GGAGGCTCAGAACCAAGAGG	GATGAGCTCCTCCCGCATC
**qRT-8400**	GGACCAGGCTTTATGTGGAGA	ACCACTCCAAGATTAGCCGA
**qRT-ACTIN2**	GGTAACATTGTGCTCAGTGGTGG	AACGACCTTAATCTTCATGCTGC
